# Clinical Outcomes of Single vs. Two-Strain Probiotic Prophylaxis for Prevention of Necrotizing Enterocolitis in Preterm Infants

**DOI:** 10.3389/fped.2021.729535

**Published:** 2021-08-30

**Authors:** Archana Priyadarshi, Gemma Lowe, Vishal Saddi, Amit Trivedi, Melissa Luig, Mark Tracy

**Affiliations:** ^1^Westmead Hospital Neonatal Intensive Care Unit, Sydney, NSW, Australia; ^2^Grace Centre for Newborn Intensive Care at The Children's Hospital at Westmead, Sydney, NSW, Australia; ^3^Department of Pediatrics, Bankstown Hospital and Sydney Children's Hospital, Sydney, NSW, Australia

**Keywords:** probiotic prophylaxis, preterms, necrotizing enterocolitis, single strain probiotic, two-strain probiotic

## Abstract

**Background:** The administration of live microbiota (probiotic) via enteral route to preterm infants facilitates intestinal colonization with beneficial bacteria, resulting in competitive inhibition of the growth of pathogenic bacteria preventing gut microbiome dysbiosis. This dysbiosis is linked to the pathogenesis of necrotizing enterocolitis (NEC), an acquired multi-factorial intestinal disease characterized by microbial invasion of the gut mucosa, particularly affecting preterm infants. Probiotic prophylaxis reduces NEC; however, variations in strain-specific probiotic effects, differences in administration protocols, and synergistic interactions with the use of combination strains have all led to challenges in selecting the optimal probiotic for clinical use.

**Aim:** To compare any differences in NEC rates, feeding outcomes, co-morbidities in preterm infants receiving single or two-strain probiotics over a 4-year period. The two-strain probiotic prophylaxis was sequentially switched over after 2 years to the single strain probiotic within this 4-year study period, in similar cohort of preterm infants.

**Methods:** During two consecutive equal 2-year epochs, preterm infants (<32 weeks and or with birth weight <1,500 g) receiving two-strain (*Lactobacillus acidophilus* and *Bifidobacterium bifidum*) and single strain (Bifidobacterium breve M-16 V,) probiotic prophylaxis for prevention of NEC were included in this retrospective, observational study. The primary outcome included rates of NEC; secondary outcomes included prematurity related co-morbidities and feeding outcomes. Time to reach full enteral feeds was identified as the first day of introducing milk feeds at 150 ml/kg/day.

**Results:** There were 180 preterm infants in the two-strain, 196 in the single strain group from the two equal consecutive 2-year epochs. There were no differences in the NEC rates, feeding outcomes, all-cause morbidities except for differences in rates of retinopathy of prematurity.

**Conclusion:** In our intensive-care setting, clinical outcomes of single vs. two—strain probiotic prophylaxis for prevention of NEC were similar. Although our study demonstrates single strain probiotic may be equally effective than two-strain in the prevention of NEC, small sample size and low baseline incidence of NEC in our unit were not sufficiently powered to compare single vs. two-strain probiotic prophylaxis in preventing NEC. Further clustered randomized controlled trials are required to study the effects of single vs. multi-strain probiotic products for NEC prevention in preterm infants.

## Introduction

Prophylactic probiotic supplementation is one extensively studied intervention in preventing Necrotizing enterocolitis (NEC), an acquired intestinal disease particularly affecting preterm infants characterized by intestinal inflammation, ischemia, necrosis, and disruption of bowel wall mucosal integrity (1). In NEC, microbial invasion of the mucosa and submucosa leads to gas production (pneumatosis intestinalis), and severe cases rapidly progress to intestinal gangrene, perforation, septicemia leading to complications such as short-gut syndrome, neurocognitive impairment, and even death (2). Multi-factorial etiology such as intestinal immaturity, inherent genetic predisposition, hypoxia-burden from cardio-respiratory disease, pathogenic microbial intestinal colonization, and poorly regulated immune responses have all been implicated leading to challenges in risk prediction and prevention of NEC (3). Preterm infants born <32 weeks or those with birth weight <1,500 g are most at risk of NEC, and the use of exclusive human milk with avoidance of cow's milk-based formula has shown to reduce NEC (4–8). A major contributing factor in the etiology of NEC is thought to be dysbiosis of the gut microbiome (7, 8). A strategy to prevent this dysbiosis is to administer live microbiota supplements to preterm infants *via* enteral route to facilitate intestinal colonization with beneficial bacteria that competitively inhibit the growth of pathogenic bacteria, altering the risk of NEC and reducing the systemic inflammatory response associated with this condition (9–12). Furthermore, probiotics have antioxidant properties, improving feed tolerance (13).

The efficacy of different probiotic strains in preventing NEC has been described. However, due to variability of the strains used and differences in administration protocols, the clinical decision on the choice of probiotics remains challenging (14–16). Probiotic products containing more than one probiotic strain have been reported to be beneficial due to their synergistic effects (17, 18). Two recent network meta-analysis of clinical trials of probiotics in preterm infants have found more evidence to support benefit of multiple strain probiotics than single strain probiotics (19, 20).

*Lactobacilli* forms a minor component of the intestinal microbiota in preterm infants. Thus, two-strain probiotic (*Lactobacillus acidophilus* and *Bifidobacterium bifidum)* offers benefit as both strains produce lactic acid and short-chain fatty acids lowering intestinal pH, inhibiting the growth of *Escherichia coli*, a common organism implicated in the pathogenesis of NEC (21).

Overall, *Bifidobacterium* when administered to preterm infants has been associated with improved weight gain, decreased intestinal permeability, reduction in the abundance of potentially pathogenic bacteria colonizing the gut (17).

However, strain matters. A specific strain of *Bifidobacterium-Bifidobacterium breve* M-16V has been shown to result in significant reduction in established (stage II) NEC, albeit a well-designed randomized controlled trial of a different *Bifidobacterium breve* strain BBG-001, showed no benefit in NEC reduction (22–27).

This study compares the NEC rates, feeding outcomes, co-morbidities in a similar cohort of preterm infants switched over from two-strain to single probiotic strain prophylaxis for NEC prevention over two equal consecutive 2-year epochs.

## Materials and Methods

A retrospective, observational study in a tertiary neonatal intensive care unit (NICU) following routine clinical practice since 2014 to supplement probiotic prophylaxis to preterm infants after parental consent as a NEC prevention intervention. These probiotic products are imported and not manufactured in Australia; due to availability issues the two-strain probiotic was switched over to the single strain.

The study eligibility criteria included all infants admitted to our NICU from July 1, 2014–July 31, 2018 and those <32 weeks and or birth weight of <1,500 g, receiving supplemental probiotic prophylaxis until completion of corrected 34 weeks. Any infants with known chromosomal abnormality and significant congenital anomaly were not included in the study.

**Figure 1:**
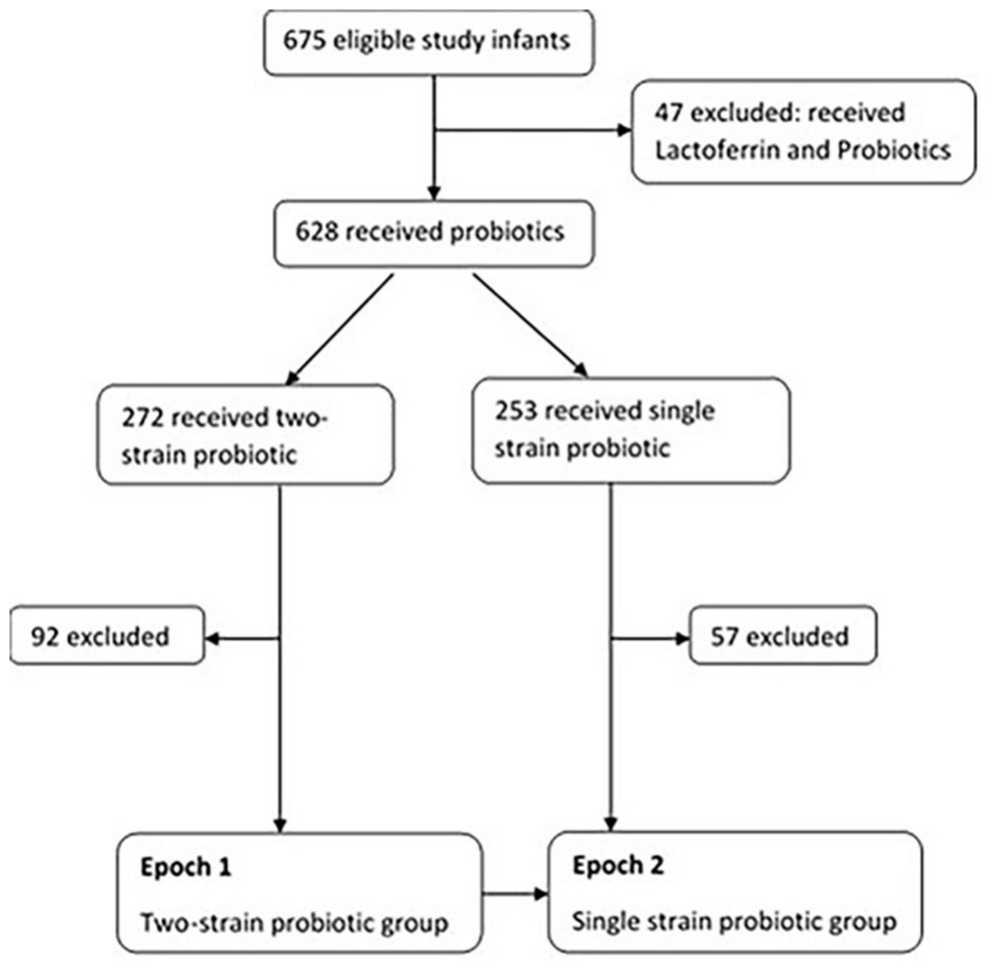
Study design.

Of the total (*n* = 675), 47 receiving probiotics and Lactoferrin (as part of another study) were excluded due to potential influence of Lactoferrin on rates of sepsis. Amongst the remaining 628 infants, 272 received two-strain and 253 received single strain probiotic supplementation. Infants that were noted to have received both the probiotics during their admission, and those transferred to other special care nurseries were excluded (*n* = 92 in two-strain and *n* = 57 in single strain) Figure 1.

**Epoch 1:** 2 years (July 1, 2014 to August 31, 2016), eligible infants (*n* = 180) received two-strain *Bifidobacterium Bifidum* & *Lactobacillus acidophilus* (Infloran® SIT Laboratorio Farmaceutico, Mede, Italy); 250 mg capsules each containing 10^9^ colony forming units (CFU) of *Lactobacillus acidophilus* 1 × 10^9^ CFU (ATCC 4356) and *Bifidobacterium bifidum* 1 × 10^9^ CFU (ATCC 15696).

**Epoch 2:** 2 years (September 1, 2016 to July 31, 2018) eligible infants (n=196) received single strain *Bifidobacterium breve* M-16 V (*Bifidobacterium breve* M-16V; Morinaga Milk Industry Co., Ltd, Japan 1 × 10^9^ colony-forming unit).

A 2-week transition period was allowed for infants receiving the two-strain probiotic to allow completion of the prophylaxis until corrected 34 weeks; infants that continued to then receive single strain, after having received the two-strain probiotic, beyond the transition period were excluded.

The primary outcome was rate of NEC between the two probiotic groups. Baseline NEC rates in our unit range from 2–6% in preterm infants < 28 weeks. The diagnosis of NEC was as per the case definition by the Australian and New Zealand Neonatal Network, ANZNN. (Diagnosis of NEC must have at least one of the following: (1). Diagnosis at surgery or post-mortem; (2). Radiological diagnosis, a clinical history plus: Pneumatosis intestinalis, or Portal vein gas, or persistent dilated loop on serial X-rays; (3). clinical diagnosis, a clinical history plus abdominal wall cellulitis, palpable abdominal mass).

Secondary outcomes included: feeding outcomes and all-cause morbidity until discharge or death during admission. The time to reach full enteral feeds was the first day of the infant receiving 150 ml/kg/day of enteral milk feeds. No changes in feeding practices, total parental nutrition use, respiratory management, or antibiotic treatment guidelines occurred during the study period.

## Probiotic Protocol

Eligible infants received freshly reconstituted contents of the probiotic every day in sterile water via enteral route and continued until the completion of corrected age 34 weeks. The probiotics were commenced soon after birth (within 12–72 h) irrespective of the feeds, after obtaining parental consent.

The two-strain probiotic (each 250 mg capsule of two-strain containing *Lactobacillus acidophilus* [10^9^ colony-forming units, NCDO 1748; National Collection of Dairy Organisms] and *Bifidobacterium bifidum* [10^9^ colony-forming units, NCDO 2203; National Collection of Dairy Organisms, Reading, United Kingdom]; Laboratorio Farmaceutico, Italy), was administered in the dose of: infants with birth weight ≥ 1 kg: 1 capsule (250 mg) and ½ capsule (125 mg) to those with birthweight <1 kg; in between feeds.

The single strain (each sachet with 1 gm dry powder containing *Bifidobacterium breve* M-16V 5 × 10^9^ colony-forming units, Bifidobacteria B. breve M-16V® Morinaga) administered in the dose of 1 ml (2.5 × 10^9^ colony-forming units) of the reconstituted solution daily *via* the enteral route, after dissolving the contents of one sachet in 2 ml of sterile water.

## Ethical Considerations

Sydney Children's Hospitals Network Human Research Ethics Committee: 2019/ETH09863, approved the study along with a waiver of consent.

## Statistical Considerations

**Figure 2:**
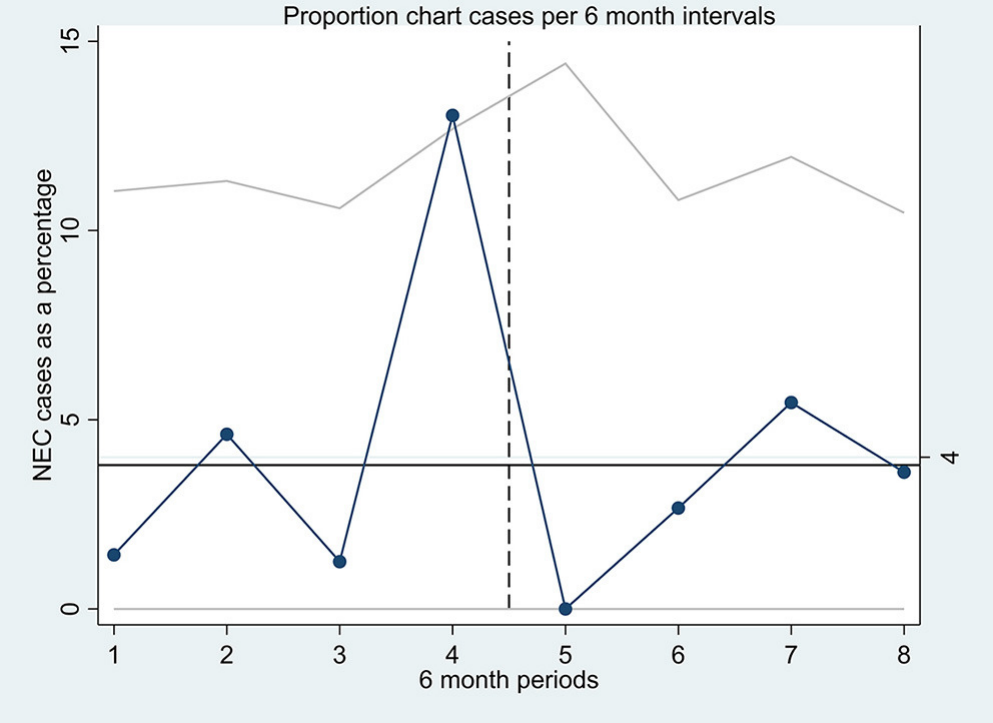
A quality assurance proportion chart (P-chart) with upper confidence interval, each point on the P-chart represents the proportion of NEC cases (in percentage) of all the infants receiving probiotics in each 6-month period of the two epochs.

Data were analyzed using Stata (V.15 MP, StataCorp, College Station, Texas, USA). Categorical variables were expressed in frequency as a number of episodes (percentage). Continuous variables were expressed as mean with standard deviation for data with a normal distribution. The two-sample *t*-test with unequal variances or Mann–Whitney test was carried out to evaluate differences in continuous variables between the two study groups. The chi-squared test was used to study the differences in proportions in categorical variables. The Fisher exact test was used where cell numbers were small. Statistical significance was considered with a *p*-value was < 0.05. Given the potential for bias arising from analysis of the retrospective cohort, logistic regression analysis to adjust for confounding variables was not performed. A quality assurance chart “Proportion chart” with upper confidence interval was created identifying the NEC cases during each 6-month period during each epoch (Figure 2).

## Results

During the study period, 376 preterm infants were identified; 180 received two-strain and 196 received single strain probiotic. The mean gestational age (28 weeks) was similar in the two groups. The infants in two-strain group had statistically significant smaller mean birth weight (1,150 g vs. 1,230 g), were small for gestational age (36 vs. 17) and had an additional day with umbilical catheter (Table 1).

**Table 1:** Clinical characteristics of preterm infants in the single vs. two-strain probiotic group.

	**Two-strain** **(** ***n*** **= 180)**	**Single strain** **(** ***n*** **= 196)**	***P*** **-value**
Gestational age (weeks)	28.3 ± 2.5	28.6 ± 2.27	0.07
Birth weight (grams)	1,151 ± 337.5	1,237 ± 363	0.01
Male, *n* (%)	90 (50%)	111(56%)	0.20
Apgar 5 min	7 ± 1.69	7 ± 1.56	0.72
SGA^*^, *n* (%)	36 (20.1)	17 (8.6)	0.002
**Mode of delivery**			
Vaginal delivery, *n* (%)	66 (37%)	79 (40%)	
Caesarian section, *n* (%)	113 (63%)	117 (60%)	0.50
**Antenatal steroids**			
No antenatal steroids, *n* (%)	23 (13%)	18 (9%)	
Antenatal steroids <24 h, *n* (%)	50 (28%)	69 (35%)	
Antenatal steroids >48 h, *n* (%)	107 (59%)	109 (56%)	0.26
Abnormal umbilical artery Doppler flows, *n* (%)	4 (2%)	13 (6%)	0.04
Umbilical artery catheter days, mean (range)	1.96 (0–9)	0.92 (0–9)	0.0003
Umbilical venous catheter days, mean (range)	1.84 (0–8)	1.06 (0–9)	0.0002

* *SGA,small for gestational age*.

There was no difference in the mean timing of commencing trophic feeds (day 2) and volume of feeds achieved by day 5 of age. The single strain group were quicker to reach full feeds (13.2 vs. 15 days). The group that received two-strain probiotic had greater number of days receiving total parental nutrition peripherally (Table 2).

**Table 2:** Feeding outcomes of preterm infants in single versus two-strain probiotic group.

	**Two-strain** **(** ***n*** **= 180)**	**Single strain (** ***n*** **= 196)**	***P*** **-value**
Day of first feed commencement	2.28 (±1.66)	2.17 (±1.27)	0.44
**Type of milk**			
Expressed breast milk (EBM)	134 (74.5%)	155 (79%)	
Formula	38 (21%)	35 (18%)	
Both formula and EBM	8 (4.5%)	6 (3%)	0.53
Volume of starting trophic feeds mL (SD)	1.2 (±1.6)	1.5 (±3.5)	0.19
Volume of feeds achieved at 72 h ml	11.5 (±19)	10.9 (±18)	0.75
Volume of feeds achieved on day 5 (ml/kg/day)	27.2 (±35)	31.4 (±34)	0.23
Days to reach 120 ml/kg/day, ± SD	15.1 (±10)	13.2 (±8)	0.04
Days to reach 150 ml/kg/day, ± SD	17.4 (±11)	15.0 (±9)	0.06
Days to reach 24 kcal/30 ml feeds, ± SD	18.5 (±14.8)	16.9 (±15)	0.30
Number of days probiotic withheld, ± SD	1.9 (±3.8)	1.3 (±24)	0.048
Number of days of centrally administered TPN*, ± SD	14.6 (±13.5)	11.7 (±11.4)	0.02
Number of days of peripherally administered TPN*, ± SD	1.9 (±3.2)	3.4 (±3.3)	<0.0001
Days to reach birth weight, ± SD	11.6 (±5)	11.7 (±5)	0.78
Total weight gain before discharge (grams), ± SD	1,532 (±800)	1,555 (±920)	0.79
weight at discharge (grams), ± SD	2,640 (±668)	2,736 (±673)	0.16

There was no statistical difference in the number of cases with suspected or confirmed cases of NEC in the two groups. There were no atypical cases of NEC, that is, NEC prior to 10 days of life or after 36 weeks postmenstrual age. There was no difference in the number of sepsis episodes, total ventilation days, number of packed red blood cell transfusion treatments, rates of intra-ventricular hemorrhage, and hemodynamically significant patent ductus arteriosus in the two groups. Rates of retinopathy of prematurity differed significantly in the two groups (Table 3). *Coagulase-negative Staphylococcus aureus* was the most common organism identified during the sepsis episodes, there were no cases of probiotic-induced sepsis.

**Table 3:** Comparison of the co-morbidities associated with prematurity in infants in the single versus two-strain probiotic group.

	**Two-strain (** ***n*** **= 180)**	**Single strain (** ***n*** **= 196)**	***P*** **-value**
Early onset sepsis ≤ 7 days, *n* (%)	0	1 (0.5%)	1.0
**Late onset sepsis > 7 days**, ***n*****(%)**			
1 episode	15(8.3%)	19(9.7%)	
≥ 2 episodes	0	3 (1.5%)	
Coagulase negative *Staphylococcus aureus*	11 cases	15 cases	
*Escherichia coli*	2 cases	0	
*Bacillus cereus*	0	1 case	
*Enterococcus faecalis*	1 case	2 cases	
*Candida albicans*	0 case	1 case	
Group B *Streptococcus*	0 case	1 case	
*Lactobacillus lactus*	1 case	0 case	
Invasive ventilation days, *n* (%)	2.73 (± 7.13)	1.67 (± 3.95)	0.08
CPAP days*, n (%)*	27.6 (± 29.9)	31.3 (± 51.18)	0.39
**Retinopathy of prematurity (ROP)** ***n*** **(%)**			
No ROP	121 (67%)	160 (82%)	0.003
Stage 1	17 (9.4%)	5 (2.5%)	
Stage 2	32 (17.8%)	21 (10.7%)	
Stage 3	10 (5.5%)	10 (5.1%)	
Treatment for ROP	10 (5.5%)	10 (5.1%)	
**Number of blood transfusion episodes**, ***n*****(%)**			
None	81 (45%)	99 (50.5%)	
≥ 1	99 (55%)	97 (49.5%)	
Transfusions (mean) (SD)	2.3 (3.4)	1.7 (2.5)	0.05
**Intraventricular hemorrhage (IVH)**, ***n*****(%)**			
No IVH	172 (95.6%)	182 (95%)	0.21
Grade 1	0	2 (1%)	
Grade 2	5 (2.8%)	8 (4%)	
Grade 3	2 (1%)	0	
Grade 4	1 (0.5%)	4 (2%)	
**Patent Ductus Arteriosus (PDA), n (%)**			
None	119 (66%)	140 (71.4%)	0.27
Hemodynamically significant PDA	61 (34%)	56 (28%)	
**NEC**, ***n*****(%)**			
None	169 (93.9)	188(95.9)	0.54
Stage 1: suspected NEC	4 (2.2%)	2 (1%)	
Stage 2: medical NEC	7(3.9%)	5 (2.6%)	
Surgical NEC	0	1 (0.5%)	
Death	4 (2%)	2 (1%)	-

There were four deaths in the two-strain strain probiotic group and two in the single strain group. The causes of death included sepsis (four cases), a severe chronic lung disease with respiratory failure (one case), and congenital dyserythropoietic anemia (one case).

## Discussion

There was no difference in NEC rates, time to reach full enteral feeds, sepsis, all-cause morbidities except for retinopathy of prematurity (ROP) rates in our study of preterm infants receiving single or two-strain probiotic prophylaxis for NEC prevention during the two consecutive equal epochs. Prophylaxis options between single or two-strain probiotic choices were restricted due to the non-availability of the two-strain product in the second epoch, and thus single strain product was used. Lack of clarity on which probiotic product to choose from once both products were subsequently available led to the study question. Both the probiotics contained a strain of *Bifidobacterium*, but they were not the same species or strain. During the study period, infants achieved similar feeding outcomes, with no difference in NEC rates, and no adverse events from probiotic-induced sepsis. Thus, it is reasonable that further recommendation on the decision of probiotic product choice is guided by other relevant factors such as ease of administration, safe storage, and cost-effectiveness in the setting of our NICU.

The mean gestational age of all study infants was 28 weeks; however, two-strain probiotic group had smaller mean birthweight and more small-for-gestational age infants with resultant longer duration of umbilical catheter and centrally administered total parenteral nutrition (TPN) days compared to the single strain group. Despite these differences, there was no difference in their rates of NEC, feeding outcomes, prematurity related co-morbidities when receiving the single or two-strain probiotic. Given the uncontrolled nature of this retrospective cohort analysis, it was not appropriate to apply multivariate logistic regression to adjust for chance differences in these baseline characteristics.

Probiotics reduce time to achieve full enteral feeds and improve feed tolerance, particularly in exclusively breastfed infants (25, 26). In our study, most infants were exclusively breastfed (74.5 vs. 79%), and human milk, formula, mixed feeding (both human milk and formula) rates were similar in both groups. There was no difference in time to reach full enteral feeds or full fortification of feeds at 24 Kcal/30 ml by adding a human milk fortifier. The time to reach full enteral feeds ranged between 15 and 17 days in both the groups, comparable to other tertiary neonatal units with a low incidence of NEC (25). During this period, access to a donor milk bank was not available. All infants regained their birth weight on day 12 and achieved a similar weight gain of about 1,500 g upon discharge. Probiotic supplementation was withheld temporarily for 2 days and 1½ days in the two-strain and single strain groups respectively, due to feeding intolerance.

Probiotics reduce risk of late-onset sepsis and NEC (12, 28, 29). Potential mechanisms include increased barrier to bacterial migration across the intestinal mucosa, competitive exclusion of potential pathogens, modification of host responses to microbes, up-regulation of immune responses by augmentation of immunoglobulin A mucosal responses, enhancement of enteral nutrition (3, 12). There was no difference in the rates of NEC or late-onset sepsis in our groups. There was no difference in the use of antibiotics between the two groups, Coagulase-negative *Staphylococcus aureus* was the most common isolated organism on blood cultures amongst infants with sepsis, there were no cases of probiotic-related sepsis.

Probiotics have no reported benefit in the prevention of retinopathy of prematurity (ROP) (30, 31). In our single strain group, preterm infants had a favorable outcome for ROP with no ROP in 82% compared to 67% in the two-strain group. Between 2017 and 2018, several quality improvement projects for reducing ROP were implemented in our NICU, such as pulse-oxygen saturation target auditing, this coincided with the phase of switching over prophylaxis to the single strain. It is plausible that the difference in ROP rates amongst the two groups may be a chance finding due to its multi-factorial etiology linked to nutrition, oxygen administration, fluctuations in pulse oximetry oxygen saturations.

Its retrospective nature is a major limitation of our study. Firstly, the small sample size was not sufficiently powered to compare NEC rates. The overall low incidence of NEC (2–6% in preterm infants <28 weeks) in our unit will require a multi-centered randomized control trial of more than 1,000 preterm infants to prove the benefit of reducing NEC. We did not report on any product quality checks to assure the effectiveness of the probiotic product when administered. We did not establish evidence on intestinal colonization post-administration of probiotics. The concept of trans-colonization during the period of change from single to the two-strain probiotic was not investigated. Given the clear evidence of cross-contamination with probiotic organisms, cluster randomized cross-over multi-center trials are required. The impact of antenatal administration of maternal antibiotics, and effects of probiotic volume on the gut osmotic load, stool microbiota were not included in our study. Finally, we did not include any long-term neurodevelopmental outcomes of infants receiving these probiotics.

In our study, strain-specific effects of probiotics did not differ in the prevention of NEC or severe stage of this disease. In addition, our study showed no differences in the clinical efficacy between the single or two-strain probiotic prophylaxis for NEC prevention in preterm infants with gestational age <32 weeks and or birth weight of <1,500 g. Based on the findings of our study, it is plausible that a single strain may be equally effective as a two-strain probiotic product. This calls for further investigations, as the impact of probiotics on incidence of NEC may not be strain-specific, and the mechanisms may be spread across the two products.

## Conclusions

In our intensive-care setting, clinical outcomes of single vs. two-strain probiotic prophylaxis for prevention of NEC were similar. Although our study demonstrates single strain probiotic may be equally effective than two-strain in the prevention of NEC, small sample size and low baseline incidence of NEC in our unit were not sufficiently powered to compare single vs. two-strain probiotic prophylaxis in preventing NEC. Strain-specific effects need further exploration with cluster randomized cross-over multi-center trials adequately numbered to study the effects of different strains in NEC prevention along with a microbiome study of the stool specimens to determine the beneficial effects of intestinal colonization in improving feeding and NEC prevention in our preterm population.

## Data Availability Statement

The original contributions presented in the study are included in the article/supplementary material, further inquiries can be directed to the corresponding author/s.

## Ethics Statement

The studies involving human participants were reviewed and approved by Sydney Children's Hospitals Network Human Research Ethics Committee. Written informed consent from the participants' legal guardian/next of kin was not required to participate in this study in accordance with the national legislation and the institutional requirements.

## Author Contributions

AP: data collection, manuscript drafting, critical review of the manuscript, and statistical analysis of the data. GL: data collection and manuscript drafting. VS, AT, and MT: statistical analysis and critical review of the manuscript. ML: critical review of the manuscript. All authors contributed to the article and approved the submitted version.

## Conflict of Interest

The authors declare that the research was conducted in the absence of any commercial or financial relationships that could be construed as a potential conflict of interest.

## Publisher's Note

All claims expressed in this article are solely those of the authors and do not necessarily represent those of their affiliated organizations, or those of the publisher, the editors and the reviewers. Any product that may be evaluated in this article, or claim that may be made by its manufacturer, is not guaranteed or endorsed by the publisher.
